# LOS oligosaccharide modification enhances dendritic cell responses to meningococcal native outer membrane vesicles expressing a non-toxic lipid A

**DOI:** 10.1111/cmi.12231

**Published:** 2013-11-06

**Authors:** Hannah E Jones, Alastair Copland, Hendrik Jan Hamstra, Jonathan Cohen, Jeremy Brown, Nigel Klein, Peter van der Ley, Garth Dixon

**Affiliations:** 1 Infectious Diseases Microbiology Unit, Institute of Child Health, UCLLondon, UK; 2 Unit Vaccinology, National Institute of Public Health and the Environment (RIVM)Bilthoven, The Netherlands; 3 Centre of Respiratory Research, Royal Free and University College Medical School, Rayne InstituteLondon, UK; 4 Department of Microbiology, Camelia Botnar Laboratories, Great Ormond Street HospitalLondon, UK

## Abstract

Outer membrane vesicles (OMV) are released by many bacteria, and contain immunogenic antigens in addition to harmful inflammatory factors, like lipopolysaccharides. Chemically detoxified OMV have been used in vaccines against *Neisseria meningitidis* (Nm); however, little is known about their interaction with antigen presenting cells. In this study, we investigated the interaction of Nm OMV with human dendritic cells (DC) to gain further understanding of their biological activity. We engineered a novel serogroup B Nm that is unencapsulated (*siaD*), expresses pentacylated lipid A (*lpxL1*), hence conferring reduced toxicity, and expresses an *lgtB* oligosaccharide structure designed to target OMV to DC via DC-SIGN. We show that the *lgtB* moiety is critical for internalization of NOMV by DC. Furthermore, the *lgtB* moiety significantly enhances DC maturation, IL-10 and IL-23 production in the presence of a pentacylated lipid A. While different DC phenotypes were observed for each NOMV, this had little effect on Th1 and Th2 cell differentiation; however, *lgtB*significantly increased Th17 cell expansion in the presence of pentacylated lipid A. We believe that *lpxL1/lgtB* NOMV should be considered further as a vaccine vector, particularly considering the importance of *lgtB* in antigen uptake and further human studies on antigen-specific responses should be considered.

## Introduction

*Neisseria meningitidis* (Nm) is a major cause of meningitis and septicaemia worldwide. Effective capsular polysaccharide vaccines have been developed against serogroups A, C, W135 and Y (Jodar *et al*., 2002; Harrison, 2006). The development of a vaccine against serogroup B has been difficult as the capsular polysaccharide, which is structurally similar to human neural glycans (Finne *et al*., 1983), is poorly immunogenic, therefore alternative antigens to the capsule have been investigated. Reverse vaccinology techniques have identified a number of outer membrane proteins such as Factor H-binding protein (fHbp), Neisserial Adhesin A (NadA) and Neisserial Heparin Binding Antigen (NHBA), that can confer cross protection against a range of serogroup B strains (Pizza *et al*., 2000; Giuliani *et al*., 2006). A number of these outer membrane lipoproteins have been included in the recently licensed vaccine Bexsero® (Toneatto *et al*., 2011). This vaccine has been a major achievement for the meningococcal field however, vaccine coverage predictions show it is unlikely to protect against all meningococcal B strains, and thus far there has been no wide spread introduction of this vaccine. Therefore it is vital work continues to understand how Nm interacts with the immune system to further improve current meningococcal vaccines.

Nm naturally sheds it outer membrane releasing vesicles into the environment. These outer membrane vesicles (OMV) are representative of the bacterial cell surface, and so contain outer membrane proteins (OMP) but also toxic lipooligosaccharide (LOS). Detergent extracted OMV vaccines have been successfully used for clonal outbreaks of meningococcal B disease (Martin *et al*., 1998; Dyet and Martin, 2005) but do not induce broad serogroup B coverage since the predominant bactericidal antibody response is against the major membrane protein PorA, which has high antigenic variability. It is also thought that the process of detergent extraction to remove LOS results in depletion of lipoproteins that could be to be important for generating cross protective antibody responses. An alternative to detergent extracted OMVs is native outer membrane vesicles (NOMV), which are extracted without the use of a detergent, so LOS remains in the cell membrane along with outer membrane lipoproteins. NOMV with wild-type lipid A would be too toxic for human use, therefore one strategy has been to engineer Nm that express LOS with pentacylated lipid A (*lpxL1*) which shows reduced endotoxin activity than wild-type LOS (Fisseha *et al*., 2005). *lpxL1* NOMVs are in fact currently being investigated as potential meningococcal vaccines (Keiser *et al*., 2010; Koeberling *et al*., 2011). Interestingly *lpxL1* lipid A variants are found naturally and appear to be associated with a less severe meningococcal disease (Fransen *et al*., 2009; Rodenburg *et al*., 2012).

While the identification of the most appropriate vaccine antigens that confers broad coverage of serogroup B strains is important, the way in which these meningococcal antigens are presented in a vaccine could also be critical for the generation of effective immunity. NOMV provide a potential delivery system for multiple epitopes presented in their native conformation. In order to further aid antigen delivery it is also possible to target vaccine components to antigen presenting cells, particularly dendritic cells (DC) (Tacken and Figdor, 2011; Ueno *et al*., 2011). DC are professional antigen presenting cells that are critical for the induction of protective immunity (Banchereau *et al*., 2000). Immature DC are highly phagocytic and express an array of pattern recognition receptors (Takeuchi and Akira, 2010), which stimulates the process of DC maturation and initiates the stimulation of naïve T-cells and shapes the nature of adaptive immune response. For these reasons, DC have been considered an ideal target for vaccine antigens. One receptor that is expressed abundantly by DC of the lymphoid tissues and mucosal surfaces is dendritic cell-specific intercellular adhesion molecule-3-grabbing non-integrin (DC-SIGN) (Geijtenbeek *et al*., 2000; Soilleux *et al*., 2002). Our group and colleagues have shown that LOS structure can be modified by deletion of the *lgtB* gene revealing a structural motif that is specifically recognized by DC-SIGN (Steeghs *et al*., 2006). Furthermore, Nm expressing the *lgtB* LOS oligosaccharide modification are more readily phagocytosed by human DC than their wild-type strain predominately via DC-SIGN (Steeghs *et al*., 2006).

Despite the fact that a number of effective vaccines have been shown to be potent stimulators of DC *in vitro* (Querec *et al*., 2006; Agrawal *et al*., 2009), there are few studies that have investigated how OMVs vaccines interact with DC (Mesa *et al*., 2004; Rodriguez *et al*., 2005; Schultz *et al*., 2007). In this study, we investigated the interaction of meningococcal NOMV with human monocyte-derived DC to further understand the biological activity of NOMV in host immune interactions. We also prepared NOMV from a novel meningococcal mutant that lacks capsule (*siaD*), to avoid potential autoreactivity, expresses LOS with a pentacylated lipid A that displays reduced toxicity (*lpxL1*) and a truncated oligosaccharide (*lgtB*) that can target DC-SIGN on DC. By evaluating the induction DC maturation, phagocytic uptake, DC cytokine induction and T-cell stimulation we provide evidence that such a strategy could be used to generate a novel vaccine delivery system to protect against serogroup B meningococcal disease.

## Results

### NOMV from LOS modified strains of *N. meningitidis* induce distinct expression patterns of maturation receptors on the surface of DC

**Figure 1:**
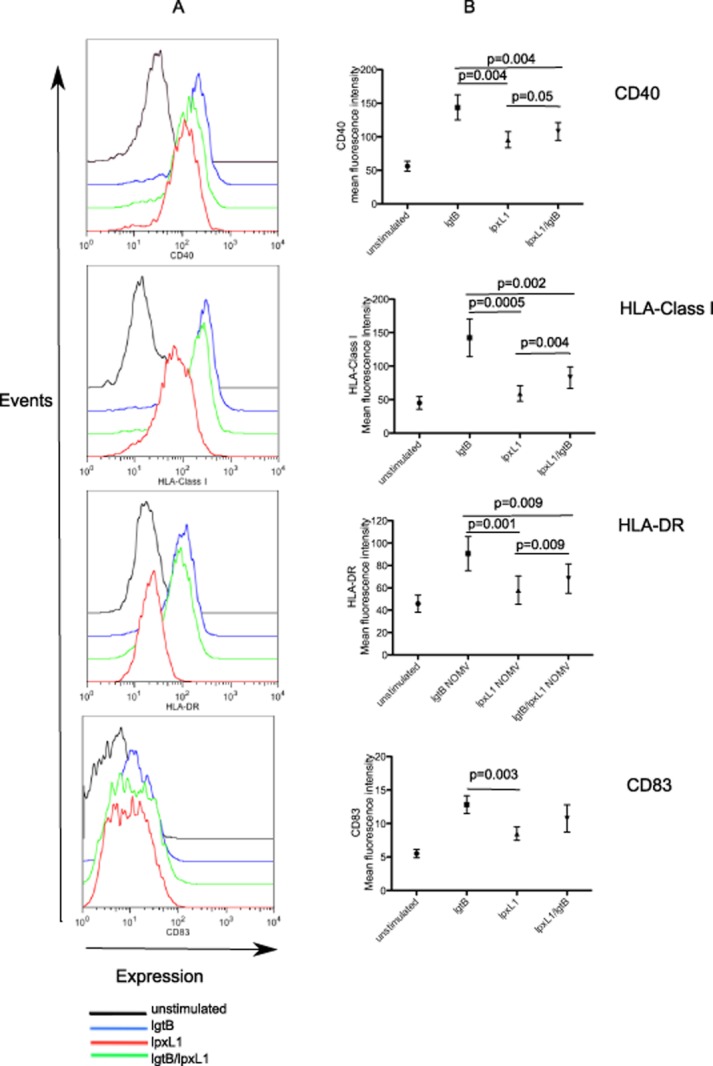
Dendritic cell surface phenotype following stimulation with LOS modified NOMV. Human monocyte-derived DC were stimulated with 1 μg ml^−1^ NOMV for 18–24 h and then analysed by Flow Cytometry for the expression of surface proteins CD40, HLA-DR, HLA-Class I and CD83. Data from one representative donor are shown (A) together with a summary of data from eight individual human donors (B). Data are expressed as the mean and standard error of the mean and significance was determined by a paired *t*-test.

Human DC were stimulated with NOMV from the LOS modified strains and surface expression of HLA-Class I, HLA-DR, CD40 and CD83 were determined by Flow Cytometry. An increase in the expression of all molecules examined was observed upon stimulation with *lgtB* NOMV when compared to unstimulated DC (Fig. 1). These were similar levels to those expected from either purified LOS or killed Nm whole bacteria. Similar expression levels were also observed with NOMV from unencapsulated wild-type bacteria (data not shown). The *lpxL1* NOMV with a pentacylated lipid A induced modest expression of CD40 compared to unstimulated but induced little to no expression of HLA-Class I and HLA-DR and CD83. This finding is consistent with reduced inflammatory potential of *lpxL1* lipid A compared to its hexacylated wild-type lipid A counterpart. However, the combination of a pentacylated lipid A and DC-SIGN recognition motif (*lpxL1/lgtB*) significantly increased the expression levels for CD40 (*P* = 0.05), HLA-DR (*P* = 0.009) and HLA-Class I (*P* = 0.004), but not CD83, when compared to those observed for the NOMV derived from the single *lpxL1* mutation. This finding demonstrates enhanced expression of certain surface markers through the engagement of DC-SIGN in the presence of a *lpxL1* mutation.

### LOS structure critically influences uptake of NOMV by DC

**Figure 2:**
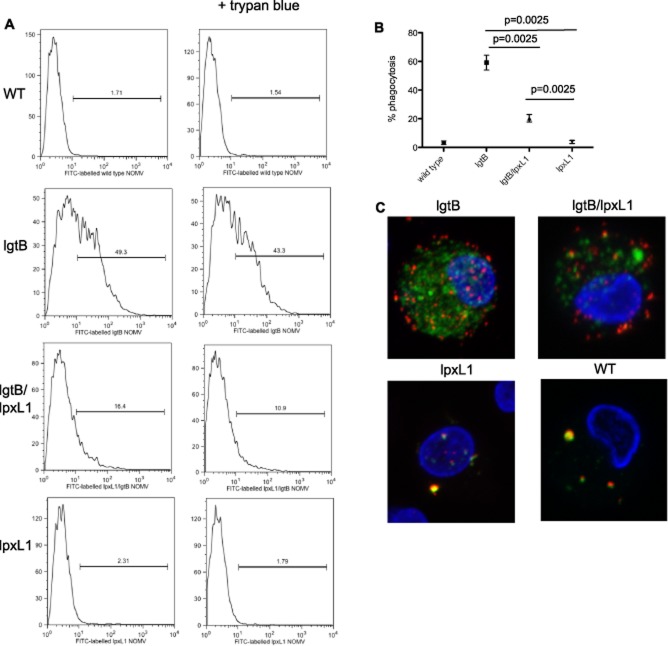
Internalization of LOS modified NOMV by dendritic cells. Human monocyte-derived DC were co-cultured with 10 μg ml^−1^ FITC labelled NOMV for 4 h. DC were separated into 3 aliquots to distinguish internalized and surface adhered NOMV. One aliquot was left untreated and the second was treated with 0.4% trypan blue to quench FITC signal emitted from extracellular NOMVs. DC were then analysed by flow cytometry. The third aliquot was stained with To-Pro3 (blue) to stain the nucleus and anti-meningococcal serosubtype P1.7 antibody followed by Alex Fluor 568 goat anti-mouse to stain the surface adhered NOMV. Cells were then analysed by Confocal Microscopy. Data are shown from one representative donor (A and C) together with the summary of data from eight donors (B). Data are expressed as the mean and standard error of the mean and significance was determined by a paired *t*-test.

*lgtB* modified Nm are more readily phagocytosed than Nm expressing wild-type LOS (Steeghs *et al*., 2006). One potential advantage of using *lgtB* modified NOMV is to increase uptake of meningococcal antigens to DC. Human DC were co-cultured with FITC-labelled LOS-modified NOMV and uptake measured by flow cytometry. As shown in Fig. 2A and B, the *lgtB* NOMV were most readily internalized by DC. In contrast, no obvious uptake was observed for the *lpxL1* NOMV even when NOMV concentrations were increased beyond 10 μg ml^−1^. Interestingly, the *lpxL1/lgtB* NOMV were taken up by DC but not to the same extent as those observed for *lgtB* NOMV. More surprisingly, NOMV from unencapsulated Nm with wild-type (WT) LOS (H44/76 *SiaD*) were not taken up by DC, displaying a very similar pattern as seen for *lpxL1* NOMV. These data strongly suggest that majority of phagocytosis is via interaction with DC-SIGN. In order to confirm further that NOMV were internalized we used a differential antibody staining and confocal microscopy method to distinguish NOMV which were either surface bound and internalized by DC. As Fig. 2C shows, both *lgtB* and *lpxL1/lgtB* NOMV are internalized, while *lpxL1* and WT NOMV were only found on the surface, thus confirming flow cytometric observations.

**Figure 3:**
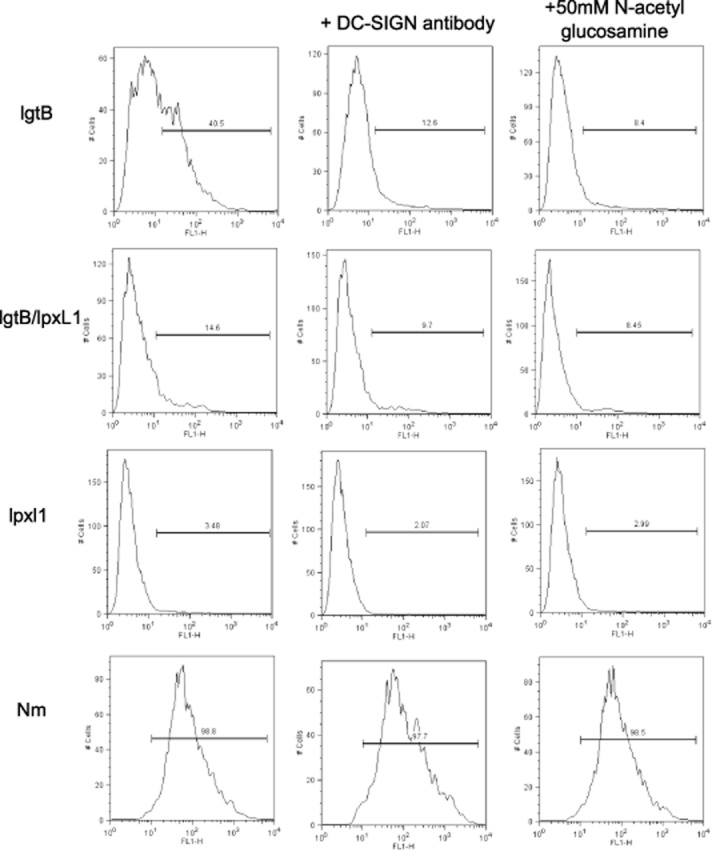
Effect of DC-SIGN blocking antibody and GlcNAc on internalization of LOS modified NOMV. Human monocyte-derived DC were stimulated with 10 μg ml^−1^ FITC-labelled NOMV for 4 h in the presence of either 20 μg ml^−1^ DC-SIGN blocking antibody or 50 mM *N*-acetyl glucosamine. DCs were then treated with 0.4% trypan blue to quench any FITC signal from surface bound NOMV. Data are representative of three experiments from three different donors yielding similar results.

We next examined the levels of phagocytosis in the presence of both DC-SIGN blocking antibody and *N*-acetyl glucosamine (GlcNAc), which have been previously shown to block the interaction between DC-SIGN and *lgtB* expressing Nm (Steeghs *et al*., 2006). Whole *N. meningitidis* bacteria expressing wild-type LOS were included as a control as its uptake is independent of DC-SIGN. As Fig. 3 shows the uptake of *lgtB* and *lpxL1/lgtB* expressing NOMV were reduced in the presence of DC-SIGN receptor blockers. Uptake was not completely prevented as a small percentage of cells were still positive for FITC although it is important to note that complete blocking of DC-SIGN even with high antibody concentrations can be difficult as DC-SIGN is so highly expressed on human monocyte derived DC. As expected, no effect of DC-SIGN blocking antibody or *N*-acetyl glucosamine was observed for the uptake of *lpxL1* NOMV (Fig. 3) or whole bacteria expressing wild-type LOS. Taken together, this shows that the majority uptake of NOMV containing *lgtB* LOS, whether on wild-type or *lpxL1* lipid A background, is via the receptor DC-SIGN.

### NOMV from LOS modified Nm modulates DC cytokine production

**Figure 4:**
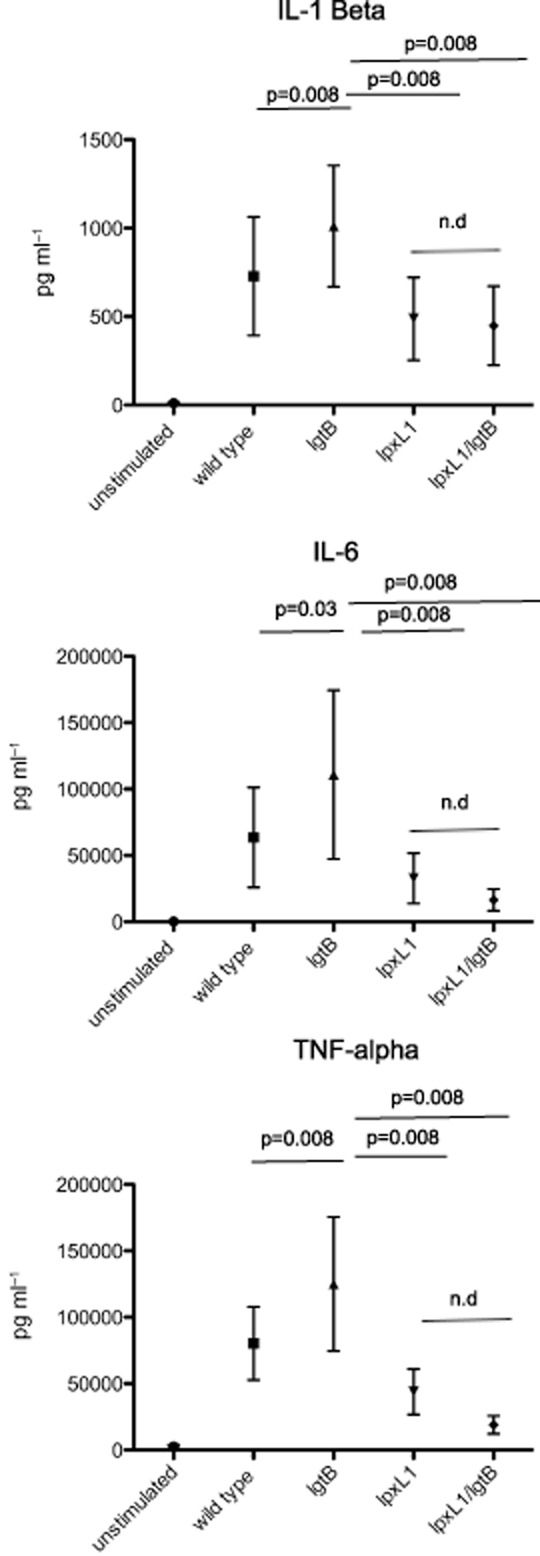
The production of inflammatory cytokines by DC in response to LOS modified NOMV. Human monocyte-derived DC were stimulated with 1 μg ml^−1^ LOS modified NOMV for 18–24 h. Supernatants were collected and then analysed for the presence of inflammatory cytokines TNF-α, IL-1β, IL-6, by ELISA. A summary of data is shown for 8 human donors. Data are expressed as the mean and standard error of the mean. Statistical significance was tested using a paired *t*-test.

Wild-type LOS is a potent stimulator of innate cells inducing a robust pro-inflammatory cytokine response, which is the major reason why DOMV, which have dramatically reduced LOS content, have been used in currently licensed vaccines. The rationale for using an *lpxL1* modified NOMV is to prevent a pronounced inflammatory host response, which could lead to excessive inflammation and toxicity. However, there are few data that are available on the effect of *lpxL1* expressing NOMV on host inflammatory responses, specifically in DCs. DCs were stimulated with LOS-modified NOMV for 18 h, and then supernatant from these cells was collected and analysed by ELISA for TNF-α, IL-6 and IL-1β production. As Fig. 4 shows *lgtB* NOMV induced the greatest amount of inflammatory cytokine production from DC, significantly more than those observed for NOMV from an unencapsulated wild-type bacteria (IL-1β, *P* = 0.008; IL-6, *P* = 0.03; TNF-α, *P* = 0.008). However, consistent with the reduced pro-inflammatory capacity of *lpxL1* LOS, both *lgtB/lpxL1* and *lpxL1* NOMV induced significantly reduced cytokine production compared to *lgtB* NOMV (*P* = 0.008). Notably, inflammatory cytokine levels in response to DOMV isolated from wild-type Nm were in a similar range to those induced by NOMV with pentacylated lipid A *lpxL1* (data not shown) despite of the fivefold higher LOS content of the latter.

**Figure 5:**
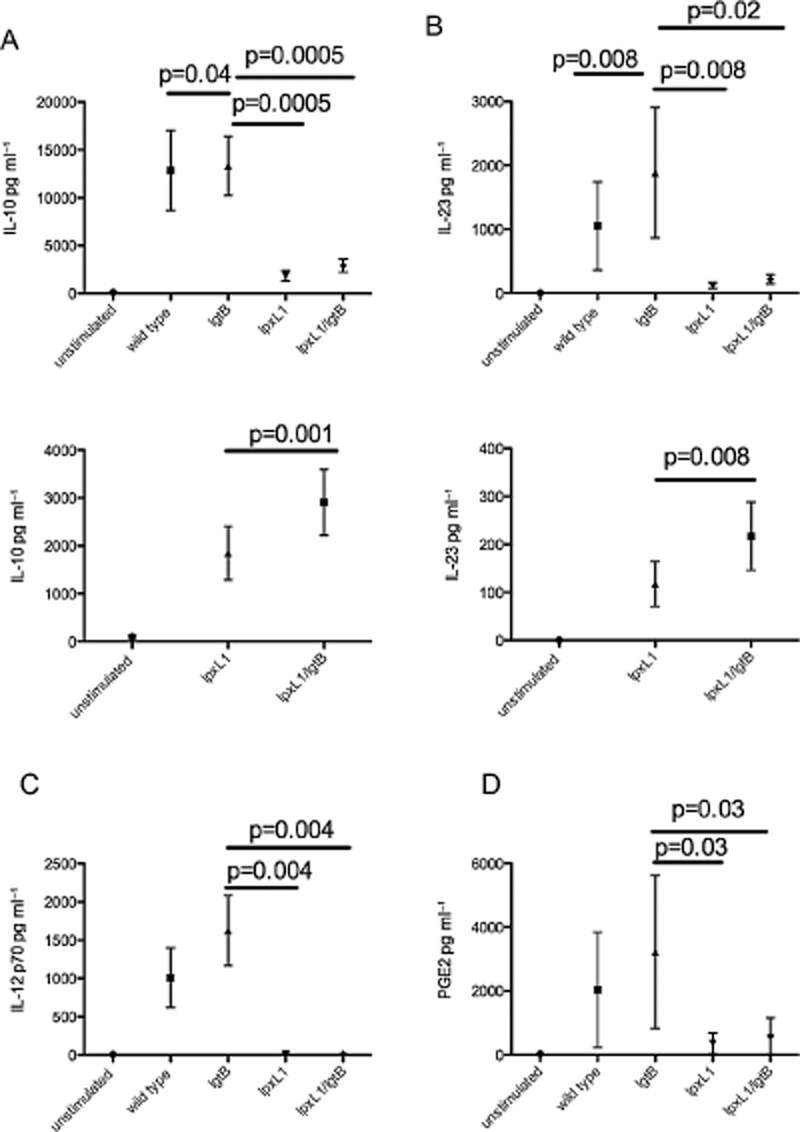
The production of T-cell polarizing cytokines by DC upon stimulation with LOS modified NOMV. Human monocyte-derived DC were stimulated with 1 μg ml^−1^ LOS modified NOMV for 18–24 h. Supernatants were collected and then analysed for the presence T-cell polarizing cytokines IL-10 (A), IL-23 (B), IL-12p70 (C) and PGE_2_ (D) by ELISA. A summary of data is shown for eight human donors. Data are expressed as the mean and standard error of the mean. Statistical significance was tested using a paired *t*-test.

We then investigated whether LOS modification of NOMV influenced the production of T-cell stimulatory cytokines IL-12, IL-10, IL-23 and also Prostaglandin E2 (PGE_2_) by DC. Since we have previously shown that uptake of whole wild-type bacteria is required for the production of both IL-10 and IL-12p70 by human DC (Jones *et al*., 2007), we hypothesized that the observed differences in uptake between the LOS modified NOMV may have a similar effect on production of IL-10 and IL-12p70. As Fig. 5 shows, IL-12p70 production by DC was detected in response to *lgtB* NOMV however no IL-12 was induced by either NOMV preparations expressing *lpxL1* pentacylated lipid A. In contrast, all NOMV preparations induced IL-10, IL-23 and PGE_2_ production, but significant reduction in levels were observed for NOMV with a pentacylated lipid A (Fig. 5: IL-10 *P* = 0.0005; IL-23 *P* = 0.008; PGE_2_ *P* = 0.03). Despite the significant reduction in IL-10 and IL-23 levels in DC stimulated with pentacylated lipid A compared to NOMV expressing wild-type lipid A, IL-10 production was still produced in the range of 6000–500 ng ml^−1^ and IL-23 500–50 ng ml^−1^ depending on donor. Moreover, for IL-10 and IL-23_,_ a significant, albeit small increase in cytokine levels was found for the *lpxL1/lgtB* NOMV compared to *lpxL1* NOMV (IL-10 *P* = 0.001; IL-23 *P* = 0.008) suggesting that *lgtB* modification on pentacylated background itself can enhance IL-10 and IL-23 production in DC (Fig. 5). There was no significant difference in the levels of PGE_2_ and IL-12 production induced by WT and *lgtB* NOMV; however, a significant reduction of IL-23 and IL-10 production was observed for WT NOMV (IL-10 *P* = 0.0391; IL-23 *P* = 0.0078) compared to *lgtB* NOMV.

**Figure 6:**
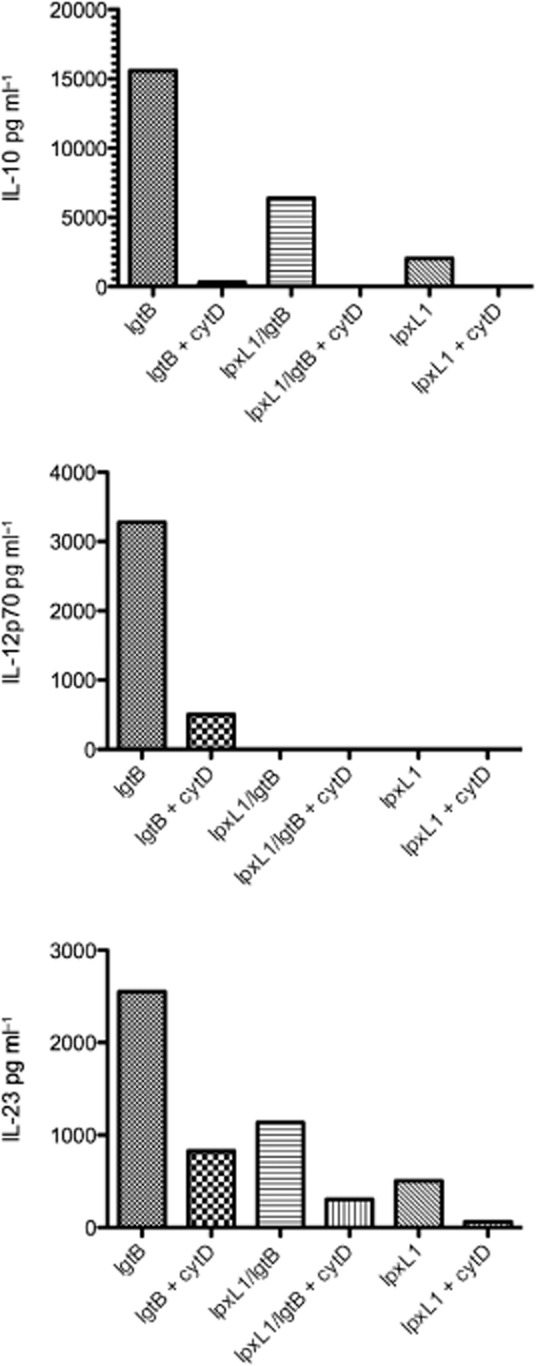
The effect of blocking phagocytosis of NOMV on DC cytokine production. Human monocyte-derived DC were co-cultured with 1 μg ml^−1^ NOMV in the presence of 10 μg ml^−1^ cytochalasin D (cytD) for 18–24 h. Supernatant was collected and analysed for IL-10, IL-12p70 and IL-23 production. Data from one representative donor out of three are shown.

We have previously shown that phagocytosis of whole Nm is indispensible for both IL-10 and IL-12 p70 production by DC (Jones *et al*., 2007), we therefore next investigated whether the uptake of NOMV also influenced cytokine production. DC were co-cultured with NOMV in the presence of the phagocytosis inhibitor cytochalasin D. As Fig. 6 shows, phagocytic uptake of NOMV also appears to be indispensible for the production IL-10 and IL-12p70 as the inclusion of cytochalasin D almost completely abrogated cytokine production of these cytokines. Interestingly, the effect of blocking phagocytosis on IL-23 production was only partial, with IL-23 levels being reduced by more than 50%. In contrast, cytochalasin D had a minimal effect on TNF-α, IL-1β and IL-6 production (data no shown) suggesting no major role for phagocytosis for induction of these cytokines, consistent with what we have demonstrated previously for whole bacterial interactions with human DC (Uronen-Hansson *et al*., 2004).

### Induction of CD4+ T helper responses by NOMV stimulated DC

**Figure 7:**
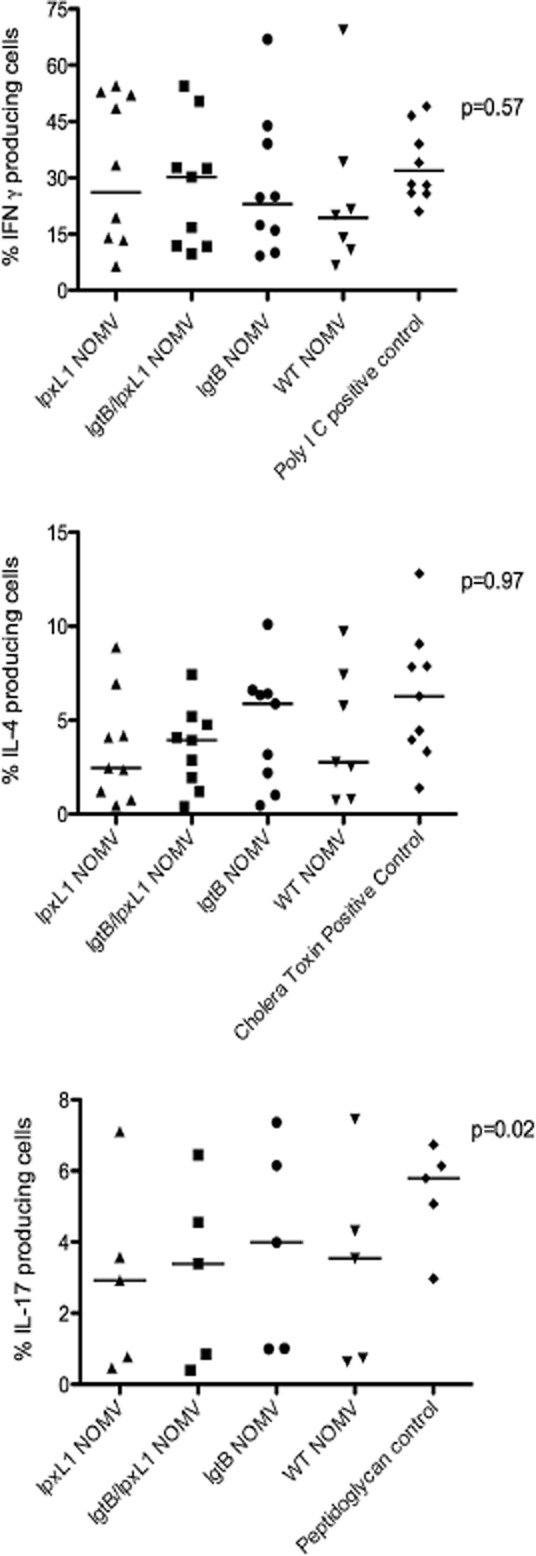
The effect of NOMV stimulated DC on Th1, Th2 and Th17 responses. To assess the generation of Th1 and Th2 responses naïve CD4+ T-cells were co-cultured with NOMV-stimulated DCs (1 μg ml^−1^ for 18–24 h) for 12 days in the presence of IL-2 and 100 pg ml^−1^ SEB. On the 12th day T-cells were restimulated with 10 ng ml^−1^ PMA and 1 μg ml^−1^ ionomycin for 5 h, cells were then analysed by flow cytometry for the production of IFN-γ and IL-4 (Th1 and Th2 skewing cytokines respectively). To assess Th17 cell responses NOMV-stimulated DC (1 μg ml^−1^ for 18–24 h) were co-cultured with memory CD4+ cells for 5 days in the presence of IL-2 and SEB. T-cells were then stimulated with PMA and ionomycin and then analysed by flow cytometry for IL-17 production. Each point on the graph represents each separate donor and the mean of each group is also shown. Statistical significance was determined by a Friedman test. Significant differences are indicated on the figure.

We next wanted to establish what, if any, functional consequence these differences in DC phenotype and cytokine profiles had on capacity to stimulate T-cells. We were particularly concerned to establish whether targeting NOMV via DC-SIGN could cause excessive skewing of naive T-cells towards a Th1 or Th2 phenotype. In the case of DC-SIGN engagement, this can often give contrasting immune effects depending on ligand, in some cases it can drive effector T-cells whereas in other instances specific pathogens use it for immune evasion (Zhou *et al*., 2006). Using an established DC and naïve CD4+ T-cell *in vitro* model system (de Jong *et al*., 2002), DC were stimulated with NOMV for 24 h and then co-cultured with naïve CD4+ T-cells for 12 days in the presence of IL-2 and SEB (Fig. 7). The proportion of Th1 and Th2 cells were identified by the expression of IFN-γ and IL-4 respectively. DC and T-cells co-cultures from nine individual donors were analysed for the presence IL-4 and IFN-γ producing T-cells by Flow Cytometry. All NOMV induced IFN-γ- and IL-4-producing cells to a similar magnitude to Th1 (Poly IC) and Th2 (Cholera Toxin) positive controls. As Fig. 7 shows, there were some observed differences in median percentage positive naïve CD4 cells producing Th1 cytokine IFN-γ, with *lpxL1/lgtB* pulsed DC promoting the greatest and the wild-type NOMV the least. As far as promoting Th2 polarized IL-4-producing cells, the *lgtB* NOMV pulsed DC promoted the most and the wild-type the least. However, these difference were not found to be significantly different (Friedman sign ranks test IFN-γ *P* = 0.57; IL-4 *P* = 0.97). Taken together, these data suggest that the *lgtB* LOS, whether with wild-type or pentacylated lipid A, does not promote significant skewing of naïve T-cells by DC.

Human DCs are also known to promote the expansion of Th17 cells from memory T-cells (van Beelen *et al*., 2007), and Th17 responses have been shown to be important for protection against mucosal pathogens by stimulating the production of anti-microbial peptides and the recruitment of neutrophils (McAleer and Kolls, 2011). Considering the role of IL-23 in promoting the expansion of Th17 cells from human memory CD4+ T-cells and the differential expression of IL-23 by DC in response to the NOMV we hypothesized that this could affect the generation of Th17 cells. NOMV stimulated DC were co-cultured with memory CD4+ T-cells for 5 days and then assessed for IL-17 production by flow cytometry (Fig. 7). Low percentages of IL-17 producing T-cells were detected; however, these are similar levels to those observed in the literature (Truchetet *et al*., 2011). Despite small differences in percentage between the groups there was a significant difference (Friedman test *P* = 0.0239). *lgtB* NOMV stimulated DC promoting the greatest percentage of IL-17 producing T-cells, with *lpxL1* NOMV stimulated DC promoting the least. These data suggest that the differential capacity of NOMV to stimulate IL-23 by DC production could be responsible for the capacity of DC pulsed with NOMV preparations to influence promotion of Th17 cells from memory T-cells.

### Immunogenicity of NOMV from LOS modified Nm in a murine *in vivo* model

**Figure 8:**
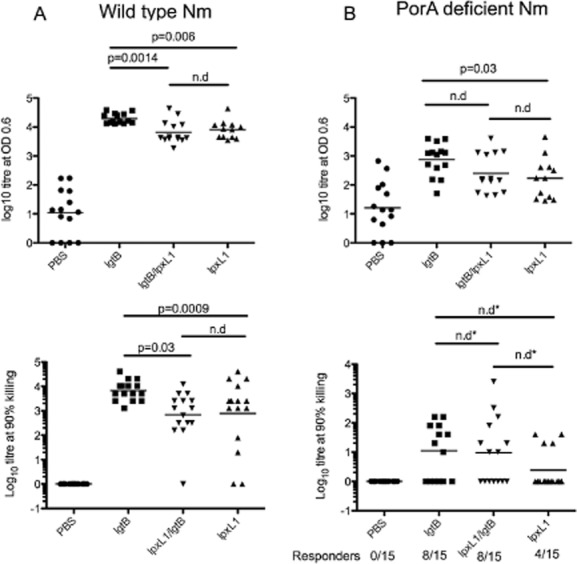
The immunogenicity of NOMV in a murine *in vivo* model. BALB/c mice (15 per group) were immunized with 5 μg of *lgtB,* *lpxL1/lgtB* or *lpxL1*. Serum was collected at day 42 and analysed for specific IgG antibody by ELISA (A). Wild-type H44/76 and PorA-deficient H44/76 were used as target antigens in the ELISA. Specific IgG levels are expressed as log_10_ titres for individual mice and the geometric mean is shown for each group. Antibody function was assessed by SBA (B). Briefly, mouse serum was incubated with rabbit complement together with either wild-type H44/76 or PorA-deficient H44/76. Highest dilution that resulted in 90% killing of bacteria is shown for individual mice and the geometric mean for each group. Statistical significance was determined by a paired T-test unless stated otherwise. For SBA against PorA-deficient H44/76 the number of responders are indicated on the graph. A responder was defined as a mouse that who serum killed 90% of bacteria at at least 1:5 dilution. *Statistical significance was determined by Fishers Exact Test.

While we have shown *in vitro* in human cells that *lpxL1* and *lpxL1/lgtB* induced significant differences in antigen internalization and the expression of specific cytokines and maturation molecules in human DC, it is unknown how this may effect the immunogenicity of the LOS-modified NOMV *in vivo*. To gain some initial immunogenicity data of the modified NOMV, mice were immunized with 5 μg of *lgtB, lpxL1, lpxL1/lgtB* on days 0, 14 and 28 and then serum was analysed for antibody by whole Nm ELISA and serum bactericidal assay (SBA) at day 42. All mice produced specific IgG to wild-type H44/76 Nm in response to the NOMV (Fig. 8A) and this was shown to have SBA against wild-type H44/76. However, there was a significant reduction in the IgG levels (*P* = 0.006, *P* = 0.0014) and serum bactericidal activity (*P* = 0.0009, *P* = 0.03) in mice that were immunized with NOMV containing *lpxL1* modification. No difference was observed between *lpxL1/lgtB* and *lpxL1* NOMV.

The antibody response to Nm is predominantly directed against the outer membrane protein, PorA (Martin *et al*., 2006), which is antigenically variable and results in a strain-specific response to Nm. We therefore wanted to determine if there was antibody to proteins other than PorA. For this we used PorA-deficient H44/76 bacteria for ELISA and SBA assays. There was a log reduction in IgG titres against the PorA-negative strain compared to wild-type H44/76 for all NOMV immunized. There was a significant difference between IgG titre generated in response to *lgtB* compared to *lpxL1* (*P* = 0.03)*,* but no significant difference was observed between *lpxL1* and *lpxL1/lgtB*. For SBA against PorA-deficient H44/76 significant differences were detected by Fishers Exact Test as not all mice responded in this assay (*lgtB* 8/15 responders; *lpxL1/lgtB* 8/15 responders; *lpxL1* 4/15 responders). No significant differences were observed between the groups; however, it is interesting to note the 8 responders in the *lpxL1/lgtB* group compared 4 in the *lpxL1* immunized group.

## Discussion

Targeting of vaccine components directly to DC has been considered an effective strategy for many years (Tacken and Figdor, 2011). In this study we have utilized the modified meningococcal *lgtB* LOS which binds to DC-SIGN (Steeghs *et al*., 2006), together with *lpxL1* LOS modification to limit endotoxin toxicity. *Lpxl1* NOMV are actively being considered as vaccines against meningococcal disease (van der Ley and van den Dobbelsteen, 2011), we therefore wanted to understand how a further *lgtB* modification may impact on human *in vitro* DC and T-cell responses. We have shown that the addition of a *lgtB* modification to a pentacylated lipid A NOMV significantly increases the upregulation of CD40, HLA-Class and HLA-DR, but does not appear to affect the reduced pro-inflammatory effect of the *lpxL1* lipid A compared to wild-type lipid A. Moreover, the addition of *lgtB* moiety appears to be critical for targeting meningococcal antigens for uptake by DC. Small, but significant differences in DC IL-23 levels also correlated with IL-17 production from memory T-cells. However despite differences in IL-10 and IL-12 production, *lgtB* binding to DC-SIGN had no major impact on the Th1 and Th2 cell polarization.

The diminished levels of DC maturation by pentacylated lipid A expressing NOMV, is consistent with previous findings that show *lpxL1* LPS to be a poor stimulator of TLR4 (Steeghs *et al*., 2008). However the addition of *lgtB* mutation resulted in a significant increase in expression of CD40, HLA-DR and HLA-Class 1 compared to *lpxL1* expressing NOMV. This shows that *lgtB* modification can augment the weak stimulatory capacity of *lpxL1* LOS, and also suggests that targeting DC-SIGN is involved in this effect. This could be a significant observation as DC-SIGN engagement in some situations can suppress DC maturation (Mittal *et al*., 2009).

Surprisingly neither the wild-type or *lpxL1* NOMV were internalized by DC, despite earlier studies showing that both wild-type and *lpxL1* whole bacteria can be internalized by DC (Uronen-Hansson *et al*., 2004; Steeghs *et al*., 2008). The addition of a *lgtB* mutation has a marked effect on NOMV uptake, even in the presence of an *lpxL1* mutation. A number of receptors have been identified for the internalization of whole Nm (Peiser *et al*., 2002; Jones *et al*., 2003; 2008; Schmitt *et al*., 2009), but DC-SIGN appears to be the major receptor involved in uptake of NOMV into monocyte derived DC. The NOMV is representative of the outer membrane of the bacteria, but the major difference is the size of the particle. NOMV are less than 150 nm and particles of this size are generally internalized via clathrin-mediated endocytosis (Xiang *et al*., 2006). Interestingly, it has been shown that DC-SIGN internalization through targeting of the carbohydrate recognition domain occurs via clathrin-coated pits which may go some way to explain why the majority of NOMV uptake was via DC-SIGN (Cambi *et al*., 2009).

The *lpxL1/lgtB* NOMV were not taken up by DC to the same extent as *lgtB* NOMV suggesting that lipid A structure is important for maximal NOMV internalization. All NOMV expressed similar levels of LOS on the surface and a monoclonal antibody to *lgtB* showed there to be no structural differences between *lgtB* and *lpxL1/lgtB* NOMV. However, meningococci expressing *lpxL1* LOS are more susceptible to antibiotics, which may suggest that the outer membrane barrier may be compromised (van der Ley *et al*., 2001) and it is tempting to suggest that this may have some effect on how LOS is presented in the bacterial outer membrane. This could affect how accessory proteins such as CD14, lipopolysaccharide-binding protein and bactericidal/permeability-increasing protein (BPI), known to be involved in LOS recognition, bind to the LOS. Interestingly BPI was shown to enhance uptake of NOMV expressing wild-type LOS by DC (Schultz *et al*., 2007). Another possible explanation for the reduced uptake of *lpxL1/lgtB* compared to *lgtB* NOMV may be the reduced TLR4 engagement of *lpxL1/lgtB* LOS. It is possible that TLR4 engagement enhances uptake of NOMV through a positive feedback loop.

Regardless of the observed differences between the *lpxL1* and *lpxL1/lgtB* NOMV in terms of DC internalization and maturation, similar pro-inflammatory cytokines TNF-α, IL-1β and IL-6 were observed for both these stimuli. Moreover, the levels of pro-inflammatory cytokines observed for *lpxL1* containing NOMV were similar of a similar magnitude to those induced by DOMV from wild-type bacteria, which are currently used for meningococcal outbreaks (data not shown). This is important as absence of excessive local and systemic inflammation is a requirement for safe vaccines, consistent with studies that have shown a clear correlation between cytokine production and vaccine reactogenicity (Gaines Das *et al*., 2004).

The finding that diminished levels of IL-10, and IL-23 and no IL-12p70 were produced by DC stimulated with *lpxL1* and *lpxL1/lgtB* NOMV compared to *lgtB* NOMV is perhaps not surprising as *lpxL1* LOS poorly activates human TLR4 (Steeghs *et al*., 2008) and that the role of TLRs in the production T-cell stimulatory cytokines IL-10, IL-12p70 and IL-23 is well established (Medzhitov *et al*., 1997; Rescigno *et al*., 1998). The level of cytokine production strongly correlates with the amount of NOMV internalization implying that receptor engagement within the DC is likely to be critical. Receptors such as TLR2, TLR4 and NOD-2, which are all implicated in IL-10, IL-12p70 and IL-23 production, are known to expressed within human monocyte-derived DC (Uronen-Hansson *et al*., 2004; van Beelen *et al*., 2007). Despite the reduction in levels of IL-10 and IL-23 induced by *lpxL1* containing NOMV compared to those with hexacylated LOS, the addition of *lgtB* modification on the *lpxL1* background significantly increased production of both these cytokines by DC.

There have been relatively few comprehensive studies on T helper cell responses to meningococci. One study demonstrated an unbiased T helper cell polarization in response to meningococci carriage (Robinson *et al*., 2002). It is likely that both Th1 and Th2 responses are important for generating immunity to meningococcus or vaccine components. Th2 is often associated with presence of IL-10 (produced by DC for example) which is important for B cell isotype switching, while Th1 responses promote production of antibody isotypes IgG2a and IgG2b which are known to be important for meningococci complement mediated killing (Hoogerhout *et al*., 1995; de Kleijn *et al*., 2001).

Since DC have a unique role in polarizing naïve T helper cells and for this reason we felt that examining the ability of NOMV pulsed DC to influence this polarization informative. Our data show that, despite differences in PGE_2_ (Th2), IL-10 (Th2) and IL-12 (Th1) production and the reduced expression of maturation markers by DC, all NOMV stimulated DC induced similar ratios of Th1 and Th2 cells *in vitro*. Interestingly, the complete abrogation of IL-12 production by *lpxL1* stimulated DC had no effect on Th1 the response. However, this finding may not be all that unexpected since other cytokines such as IL-18 and Type 1 Interferons (Sareneva *et al*., 1998) are involved in Th1 responses. However the lack of an unbiased polarization towards Th1 and Th2 does show that DC-SIGN and *lgtB* engagement in this instance does not have a major effect on T-cell polarization. This could be of interest and potential significance, as DC-SIGN engagement can result in tolerogenic DC phenotype (Mittal *et al*., 2009) a finding that we believe to be important preliminary data in justifying its use in human volunteers.

We observed significant, albeit small difference in the expansion of memory Th17 cells in response to the *lpxL1/lgtB* and *lpxL1* pulsed DC. All the NOMV stimulated DC production of IL-1β, IL-6 and IL-23, which have all been implicated in Th17 cell differentiation (van Beelen *et al*., 2007). However, IL-23 levels in DC did appear to correlate with the percentage of Th17 cells, with *lpxL1* NOMV inducing the least IL-23 production and the least percentage of Th17 cells. It is not known at present how this could influence vaccine responses, but the capacity of *lgtB/lpxL1* NOMV to induce Th17 cells may be advantageous. Th17 cells play a key role in the induction of mucosal inflammation and host protection against extracellular pathogens (van Beelen *et al*., 2007), moreover, IL-17 is involved in the recruitment of neutrophils, which is important for host defence against meningococci (Bettelli *et al*., 2008; Peck and Mellins, 2010).

The murine data presented here show that, as might be expected, presence of the wild-type LOS with the *lgtB* modification significantly enhances specific antibody production and SBA to the homologous strain in both a porA-dependent and a porA-independent manner compared to responses to *lpxL1* expressing NOMV. The likely reason for this is the greater adjuvant effect of hexacylated LOS. We considered it plausible that the difference in DC co-stimulation induced by *lpxL1/lgtB* compared to the *lpxL1* NOMV might be reflected in capacity to influence antibody production and SBA. There was some suggestion from the SBA data from a PorA-deficient Nm that there was an increased number of responders in those mice immunized with *lpxL1/lgtB* (8/15) compared to *lpxL1* (4/15). However, this was not a significant difference, and therefore the activity of the two NOMV are equivalent in this model. Although we believe these to be useful and important data to include, we feel this should be interpreted with caution with regard to predicting the likely effect in humans. The mice commonly used for *in vivo* immunogenicity studies do not express an orthologue of DC-SIGN, so the effects of *lgtB* mutation may not be fully apparent in murine studies. Human DC-SIGN transgenic mice are available; however, the use of these mice in vaccination studies may not recapitulate the biology of DC-SIGN in humans (Garcia-Vallejo and van Kooyk, 2013).

We believe that the addition of the *lgtB* moiety to *lpxL1* requires further consideration and the only way to ascertain its effects *in vivo* is to use healthy human volunteers. As the *lgtB* modification is essential for internalization of meningococcal NOMV, it is tempting to speculate that this could influence meningococcal-specific immunity induced by these vaccines. It is known that efficiency of uptake of antigen by DC, as well as co-stimulatory signals derived from adjuvant factors, is an important determinant of generation of antigen-specific immunity.

A major attraction of using NOMV is that they contain multiple meningococcal antigens in native conformation. These could be modified further to express antigens of interest, for example non-binding factor H binding mutants, plus other targets to cover invasive disease caused by Nm strains that do not express fHbp, which has been one of the major vaccine antigens considered over the last few years (Lucidarme *et al*., 2011). We propose that additional modification of the LOS to include a DC-SIGN targeting motif would increase antigen delivery to DC, with better T-cell co-stimulation than *lpxL1* preparations and has potential to enhance cross protection against serogroup B.

## Experimental procedures

All reagents were obtained from Sigma-Aldrich unless otherwise stated.

### Ethics statement

Blood samples were collected from healthy laboratory volunteers with written informed consent. The Institute of Child Health Human Tissue Act Review Board approves the use of blood in this study, from healthy adults volunteers. Consent and donations were recorded in accordance the Institute of Child Health’s Research Governance and Ethical Regulations.

### Genetic modification of *N. meningitidis* strain

All genetically modified strains used in this study were derived from wild-type group B *N. meningitidis* H44/76 (Holten, 1979). The unencapsulated (siaD) *lpxL1, lgtB* and *lpxL1/lgtB* and PorA mutants were constructed by insertional deletion of the capsulation gene *siaD* and LOS biosynthesis genes *lpxL1* and *lgtB*. All modified strains were characterized by SDS-PAGE to confirm no major differences in surface protein and LOS expression. LOS structure for each of the modified strains was confirmed using mass spectrometry and monoclonal antibodies specific to the oligosaccharide part of the LOS. NOMV from unencapsulated *siaD* bacteria are defined in this study as wild-type NOMV. All other NOMV from LOS modified strains are defined by the modified gene, *lpxL1, lgtB* and *lpxL1/lgtB*.

### Isolation and characterization of native outer membrane vesicles

Bacteria were grown overnight in Tryptic Soy Broth (Oxoid) and then pelleted by centrifugation. Bacterial pellet was suspended in 0.1 M Tris, 0.01 M EDTA pH 8.6 and agitated for 1 h to encourage release of vesicles into the supernatant. Vesicles were collected from supernatant by ultracentrifugation (150 000 *g* for 1 h) and then washed with 0.05 M Tris, 2 mM EDTA, 3% sucrose pH 8.9. NOMV were then resuspended in 0.01 M Tris pH 7.4, 3% sucrose, 0.01% thimerosal and then stored at 4°C until required. Protein content of NOMV preparations was determined using a BCA assay (Thermo Scientific) and LOS content was assessed using SDS-PAGE analysis. All NOMV concentrations used in this study are based on protein content.

For phagocytosis experiments NOMV were labelled with 0.5 mg ml^−1^ Fluorescein isothiocyanate (FITC) for 30 min at 37°C. NOMV were then washed with PBS to remove excess FITC and collected by ultracentrifugation (150 000 *g* for 1 h). FITC incorporation into the NOMV was verified by flow cytometry using a FACScalibur (BD Biosciences). No major changes in the size or density of the vesicles following FITC labelling were observed. In optimization experiments, FITC-labelled NOMV also gave similar maturation and cytokine responses in DCs to unlabelled NOMV.

### Human monocyte derived dendritic cell culture

DC were generated from monocytes as previously described (Sallusto and Lanzavecchia, 1994; Dixon *et al*., 2001). Briefly, monocytes were isolated from peripheral blood mononuclear cells (PBMC) using CD14 immunomagnetic bead separation technology (Miltenyi Biotec, Surrey, UK). CD14-positive cells were cultured for 5–7 days in RPMI with 10% fetal calf serum, 2.4 mM l-glutamine, 100 U ml^−1^ penicillin, 100 mg ml^−1^ streptomycin (all Invitrogen), 100 ng ml^−1^ GM-CSF and 50 ng ml^−1^ IL-4 (R&D systems). Immature DC were DC-SIGN-positive, CD3-negative, CD19-negative, CD25-negative and expressed low levels of HLA-DR, HLA-Class I, CD40, CD83, CD86 and CD1.

### Internalization assay

DC (5 × 10^5^ ml^−1^) were co-cultured with 10 μg ml^−1^ FITC-labelled NOMV for 4 h so to maximize visualization of NOMV uptake. In some experiments 20 μg ml^−1^ of DC-SIGN blocking antibody [R&D systems (clone 120507)] or 50 mM *N*-acetyl glucosamine was included to block receptor DC-SIGN. Complete blocking of phagocytosis was achieved by the addition of 10 μg ml^−1^ cytochalasin D, an actin polymerization inhibitor. After 4 h, DC were incubated for 10 min with 0.4% trypan blue to quench FITC signal from extracellular NOMV. DC were then washed twice with FACs buffer (PBS with 0.2% bovine serum albumin and 0.02% sodium azide) and then fixed with 4% paraformaldehyde. Cells were then analysed by flow cytometry using a FACScalibur (BD Biosciences). The DC population was defined by forward and side scatter profile and at least 5000 events were collected in this gate. NOMV uptake was defined as the proportion of cells in the DC population that were positive for FITC expression. Data were analysed by FlowJo software.

For confocal microscopy analysis DC were washed with PBS following a 4 h period of FITC-NOMV stimulation and allowed to adhere to adhesion slides for 10 min (Paul Marienfeld Gmbh and CO, Germany). Cells were then fixed with 4% paraformaldehyde. To distinguish between surface bound and internalized NOMV, surface bound NOMV were stained with 10 mg ml^−1^ P1.7 antibodies specific for Nm (NIBSC, South DCs) followed by Alexa Fluor 568 goat anti-mouse IgG (Life Technologies, UK). DC were visualized by staining the nuclei with To-Pro3 (Molecular Probes, Cambridge Biosciences, Cambridge, UK). Slides were then washed and mounted with SlowFade Gold (Life Technologies, UK) and then images were obtained using a Zeiss Confocal Laser Scanning Microscope 710. To identify internalized bacteria at least 10 optical sections (0.2–0.5 mm) spanning the entire DC were visualized by open source software program Fiji (http://fiji.sc/Fiji).

### DC surface marker expression and cytokine detection

Immature DC (5 × 10^5^ ml^−1^) were co-cultured with 1 μg ml^−1^ NOMV for 18–24 h. Cells were harvested in FACs buffer and then labelled with FITC- or phycoerythrin (PE)-conjugated monoclonal antibodies (2 μg ml^−1^) to CD40, CD83, HLA-DR and HLA-Class I (Invitrogen) for 30 min at 4°C. Cells were then washed with FACs buffer and fixed with 4% paraformaldehyde. Cells were then analysed by flow cytometry using a FACScalibur (BD Biosciences).

DC supernatants were removed following 24 h stimulation with NOMV and then analysed for the presence of TNF-α, IL-1β, IL-6, IL-10, IL-12p70, IL-10 (eBioscience) and PGE_2_ (GE Healthcare, UK) by ELISA according to manufacturer’s instructions.

### DC polarization of T helper responses

Naïve CD4+ T-cell Th1 and Th2 polarization was carried out as previously described (de Jong *et al*., 2002). First, DC were stimulated with 1 μg ml^−1^ NOMV, 20 μg ml^−1^ Poly I:C (Th1 skewing positive control), and 10 μg ml^−1^ Cholera toxin (Th2 skewing positive control) for 24 h. Cells were than washed and counted. Naïve CD4+ T-cell were isolated from PBMCs using a Naïve CD4+ T-cell isolation kit (Miltenyi Biotec) and subsequently co-cultured with stimulated-DC at a ratio of 1:4 (DC:T-cell) in the presence of 100 pg ml^−1^ staphylococcus enterotoxin B (SEB) and 10 U ml^−1^ IL-2 (R&D systems). Cells were cultured for up to 12 days and then quiescent T-cells were restimulated with 10 ng ml^−1^ PMA and 1 μg ml^−1^ ionomycin for 5 h in the presence of 10 μg ml^−1^ Brefeldin A. After 5 h, an aliquot T-cells were labelled for surface expression of CD3 with a CD3 PerCP-Cy5.5-conjugated antibody (eBioscience). Cells were then fixed with 4% PFA and then permeabilized with 0.5% saponin in order to stain for intracellular cytokines. T-cells were then labelled with antibodies to IL-4 and IFN-γ conjugated with PE and FITC (Invitrogen) respectively. Cells were then fixed and analysed by flow cytometry using a FACScalibur (BD Biosciences). Alongside this a separate aliquot of cells was also taken and labelled for caspase activity using a Vybrant FAM Poly Caspase Assay Kit. Cells that were undergoing apoptosis were caspase positive and excluded from analysis. At least 50 000 T-cells were collected as defined by CD3 expression and forward and side scatter profiles. Data were analysed using FlowJo.

To assess Th17 T-cell differentiation stimulated DC were co-cultured with allogeneic memory CD4+ T-cells. DC were stimulated with 10 μg ml^−1^ *Staphlococcus aureus* peptidoglycan as a positive control for Th17 differentiation, Poly I:C (20 μg ml^−1^) was used as a negative control. Memory CD4+ T-cells were isolated using a Memory CD4+ T-cell isolation kit (Miltenyi Biotec) and then co-cultured with stimulated-DC at a ratio of 1:10 (DC:T-cell) in the presence of 100 pg ml^−1^ SEB and 2 U ml^−1^ IL-2 for up to 5 days. Cells were restimulated as before with PMA, Ionomycin and Brefeldin A and then stained for IL-17 production using an IL-17 PE-conjugated antibody (eBioscience). Cell were analysed using flow cytometry and at least 50 000 T-cells were collected and analysed.

### Murine immunizations

All animal experiments were approved by the UCL Biological Services Committee and the Home Office (UK) under Project License PPL70/6510. All experimental procedures were in line with current UK Home Office guidelines.

Four groups of 15 female BALB/c mice aged 6–8 weeks (Charles River UK) were immunized with 5 μg of *lgtB*, *lpxL1, lgtB/lpxL1* or PBS on days 0, 14 and 28. On day 42 mice were terminally bled and sera was stored at −80°C for analysis of specific antibody by ELISA and SBA.

### Antigen-specific antibodies

Specific IgG was determined by whole-cell ELISA. Briefly, 96-well ELISA plates were coated with 100 μl per well of 0.5% paraformaldehyde fixed Nm (OD 0.05 at *A*_600_) overnight at RT. Plates were washed with ELISA buffer (PBS + 0.05% Tween 80) and then 100 μl of sera serially diluted with PBS + 0.1% Tween 80 was added to each well. After 1 h incubation at 37°C, plates were washed and then 100 μl of 1/5000 dilution of goat anti-mouse IgG-HRP was added to each well. Plates were incubated for a further 1 h at 37°C. Plates were washed and then developed 3,3′,5,5′-Tetramethylbenzidine (TMB). After 10 min the reaction was stopped with 3 M sulfuric acid and read at *A*_450_. The antibody titre was calculated as the reciprocal dilution that gave an optical density of 0.6.

### Serum bactericidal assay (SBA)

Sera was diluted 1:5 with GBSS + 0.5% BSA and then heat inactivated at 56°C for 30 min. Twofold serial dilutions were made, these were then incubated with wild-type or PorA-deficient Nm H44/76 (10^4^ cfu ml^−1^) for 15 min at RT. Following incubation baby rabbit complement was added to a final volume of 20% and left for 1 h at 37°C. Bacteria were plated on GC agar supplemented with Vitox (Oxoid) and grown overnight in a humidified incubator at 37°C 5% CO_2_. The serum bactericidal titre was defined as the reciprocal dilution that resulted in 90% bacterial killing.

### Statistical analysis

Data were analysed using paired T-test, Friedman test (a non-parametric test that ranks differences in responses of individual donors to different stimuli (NOMV), taking into account the variability often observed with human donors), or Fishers Exact Test (responders versus non-responders) using GraphPad Prism software. These are stated in the text when used. Significance was defined as *P* < 0.05.
